# Performance of the No-U-Turn sampler in multi-trait variance component estimation using genomic data

**DOI:** 10.1186/s12711-022-00743-5

**Published:** 2022-07-11

**Authors:** Motohide Nishio, Aisaku Arakawa

**Affiliations:** Institute of Livestock and Grassland Science, NARO, 2 Ikenodai Tsukuba, Ibaraki, 3050901 Japan

## Abstract

**Background:**

Multi-trait genetic parameter estimation is an important topic for target traits with few records and with a low heritability and when the genetic correlation between target and secondary traits is strong. However, estimating correlations between multiple traits is difficult for both Bayesian and non-Bayesian inferences. We extended a Hamiltonian Monte Carlo approach using the No-U-Turn Sampler (NUTS) to a multi-trait animal model and investigated the performance of estimating (co)variance components and breeding values, compared to those for restricted maximum likelihood and Gibbs sampling with a population size of 2314 and 578 in a simulated and real pig dataset, respectively. For real data, we used publicly available data for three traits from the Pig Improvement Company (PIC). For simulation data, we generated two quantitative traits by using the genotypes of the PIC data. For NUTS, two prior distributions were adopted: Lewandowski-Kurowicka-Joe (LKJ) and inverse-Wishart distributions.

**Results:**

For the two simulated traits with heritabilities of 0.1 and 0.5, most estimates of the genetic and residual variances for NUTS with the LKJ prior were closer to the true values and had smaller root mean square errors and smaller mean absolute errors, compared to NUTS with inverse-Wishart priors, Gibbs sampling and restricted maximum likelihood. The accuracies of estimated breeding values for lowly heritable traits for NUTS with LKJ and inverse-Wishart priors were 14.8% and 11.1% higher than those for Gibbs sampling and restricted maximum likelihood, respectively, with a population size of 578. For the trivariate animal model with real pig data, the estimates of the genetic correlations for Gibbs sampling and restricted maximum likelihood were strongly affected by population size, compared to NUTS. For both the simulated and pig data, the genetic variances and heritabilities for NUTS with an inverse-Wishart prior were overestimated for low-heritability traits when the population size was 578.

**Conclusions:**

The accuracies of variance components and breeding values estimates for a multi-trait animal model using NUTS with the LKJ prior were equal to or higher than those obtained with restricted maximum likelihood or Gibbs sampling. Therefore, when the population size is small, NUTS with an LKJ prior could be an alternative sampling method for multi-trait analysis in animal breeding.

**Supplementary Information:**

The online version contains supplementary material available at 10.1186/s12711-022-00743-5.

## Background

Selection of livestock is usually based on a combination of several traits of economic importance that may be phenotypically and genetically related. Multi-trait analysis was introduced in quantitative genetics by Henderson and Quaas [1]. It is based on the simultaneous evaluation of animals for several traits and makes use of the phenotypic and genetic correlations between them. Compared to analyzing each trait separately, the advantages of multi-trait analysis are an increase in prediction accuracy, statistical power and parameter estimation accuracy and decrease in trait selection bias [2–4]. In particular, multi-trait analysis can provide more accurate estimations in the case of traits with a low heritability or populations of small size [5]. Accurate estimation of variance components and functional parameters, such as heritabilities and genetic correlations, is important because prediction error variances for predicted random effects increase as the differences between estimated and true values of variance components increase [6]. Accurate estimation of multi-trait variance components that considers the genetic correlations between economically-important traits will contribute to improve the accuracy of genetic evaluation.

Restricted maximum likelihood (REML) and Bayesian analyses have become standard estimation methods in animal breeding. Patterson and Thompson [7] first developed the REML approach, which has been widely used for the estimation of multi-trait (co)variance components thanks to the availability of several programs, e.g. MTDFREML [8], VCE [9], REMLf90 [10] or ASREML [11]. From a Bayesian viewpoint, REML is considered as the mode of the joint posterior distribution of all (co)variance components, with noninformative prior densities, once the fixed effects are marginalized by translation invariance functions of the data [12]. However, since the REML estimator relies on an asymptotic distribution, the inferences are valid strictly for a sample of infinite size [13, 14]. Therefore, it is difficult to calculate reliable confidence intervals for REML-based variance component parameters [15]. An alternative to REML estimation is a full Bayesian approach through Markov chain Monte Carlo (MCMC) methods, which were introduced in quantitative genetics in the early 1990s [13, 16]. Gibbs sampling (GS) is an MCMC method that repeatedly samples from the conditional distributions of one variable when all the other variables are assumed to be known [17]. In practice, GS is frequently used because it does not require the design of a proposal distribution and the procedure is simple to program. In animal breeding programs and in the case of a single-trait model, an inverse-gamma distribution is used for a prior distribution of variance components but, in practice, such a distribution has two major problems. One is that inappropriate parameters are used to make the inverse-gamma distribution as uniform as possible [13, 16, 17], and the other is that, if small values are set to make the distribution as least informative as possible such as 0.001, the inverse-gamma distribution will show a weak peak around 0, which might result in being unintentionally informative [18]. In the case of a multi-trait analysis, the inverse-Wishart (IW) conjugate family of distributions is used as priors for the covariance matrices between traits because the IW distribution is a multivariate generalization of the inverse-gamma distribution [19]. Consequently, the IW prior is expected to have the same problems as the inverse-gamma prior.

The Hamiltonian Monte Carlo (HMC) approach has become a popular alternative MCMC method, which is based on Hamiltonian dynamics used in physics and is a Metropolis strategy for all parameters simultaneously [20]. Hoffman and Gelman [21] developed the No U-Turn Sampler (NUTS), which automatically tunes the hyperparameters required for HMC. Recently, HMC and NUTS were applied to animal breeding [22, 23]. In a single-trait analysis, Nishio and Arakawa [23] showed that NUTS performed well for the estimation of variance components, in the case of large effective sample sizes, low autocorrelations, and low skewness of posterior distributions, particularly when the heritability of the trait was low. NUTS can be implemented by probabilistic programming language, such as PyMC and Stan [24]. In addition, conjugate priors are not necessary for NUTS, thus appropriate priors other than the IW priors can be used for the covariance matrix. Therefore, NUTS that uses an appropriate prior might provide more accurate estimates of variance components and breeding values in multi-trait analysis than GS.

We have introduced two estimation methods, i.e. REML and Bayesian analysis, and two computing algorithms of Bayesian analysis, i.e. GS and NUTS. The goal of this study was to compare the performances of REML, GS and NUTS for the accuracy of the estimation of variance components and breeding values. This comparison was based on multi-trait genomic best linear unbiased prediction (GBLUP) models using simulated and real pig data.

## Methods

### Real pig data

Publicly available data including genotypic and phenotypic information on a single Pig Improvement Company (PIC) nucleus pig line were used (https://academic.oup.com/g3journal/article/2/4/429/6026060). This dataset is composed of 3534 animals with phenotypes for five traits and genotypes from the PorcineSNP60 chip ($n={64,223}$). These phenotypes were already adjusted for environmental fixed effects: sex, farm and year of birth [25]. We used three traits (T1, T2 and T3) and extracted 2314 animals for which records for these three traits were available. The T1, T2 and T3 traits used in this paper correspond to the T1, T2 and T3 traits in the publicly available PIC data. Two scenarios were applied to estimate variance components: Scenario 1 that used the full data (2314 animals) and Scenario 2 that used data from 578 animals randomly selected from the 2314 animals under the assumption that few records were available for the T1, T2 and T3 traits.

Criteria to exclude single nucleotide polymorphisms (SNPs) were: a minor allele frequency lower than 0.05, a call rate lower than 0.95 and a Hardy–Weinberg equilibrium cut-off P value lower than 0.001. After quality control, the final data set included 33,860 SNPs. In this study, we used the genomic relationship matrix as an additive genetic relationship matrix that was denoted $\mathbf{A}$ in this paper and computed according to VanRaden [26]:

1 $${\bf{A}} = \frac{{{{\bf{M}}_{\bf{a}}}{{{\bf{M'}}}_{\bf{a}}}}}{{\sum\nolimits_{j = 1}^{{N_{snp}}} 2 {p_j}(1 - {p_j})}},$$

where ${p}_{j}$ is the frequency of the second allele at SNP $j$, $\mathbf{M}$ is the ${n\times N}_{snp}$ matrix ($n$ is the number of genotyped animals and ${N}_{snp}$ is the number of SNPs) and the elements (${m}_{ij}$) of $\mathbf{M}$ for animal $i$ at SNP $j$ are calculated as ${m}_{ij}={g}_{ij}-2{p}_{j}$, where ${g}_{ij}$ (coded as 0, 1 or 2) is the number of the second allele of animal $i$ and SNP $j$.

### Simulated data

To validate the performances of REML, GS and NUTS for the estimation of variance components, we also generated a simulated dataset from the genotypes of the real PIC pig dataset. Similar to the real data, we used the genomic relationship matrix ($\mathbf{A}$) as an additive genetic relationship matrix. We simulated two quantitative traits (trait1 and trait2) by summing up the additive genetic effects $\mathbf{a}$ and the residuals $\mathbf{e}$. Thus, the vector of phenotypes was calculated as $\mathbf{y}=\mathbf{a}+\mathbf{e}$, where the $\mathbf{a}$ and $\mathbf{e}$ vectors were drawn from the multivariate normal distributions $MVN({\bf{0}},{{\bf{G}}_0}\bf{\otimes} {\bf{A}})$ and $MVN({\bf{0}},{\mathbf{R}}_{0}\bf{\otimes}\mathbf{I})$, respectively; ${\mathbf{G}}_{0}$ is the $n\times n$ additive genetic covariance matrix and ${\mathbf{R}}_{0}$ is the $n\times n$ residual covariance matrix for $n$ traits. We used the Cholesky decomposition of the covariances $\mathbf{G}(={\mathbf{G}}_{0}\bf{\otimes}\mathbf{A})$ and $\mathbf{R}(={\mathbf{R}}_{0}\bf{\otimes}\mathbf{I})$ to draw samples from the multivariate normal distribution. The random additive genetic effect $\mathbf{a}$ was calculated as $\mathbf{a}={\mathbf{L}}_{a}{\mathbf{z}}_{a}$, where ${\mathbf{z}}_{a}\sim MVN({\mathbf{0}},\mathbf{I})$ and ${\mathbf{L}}_{a}$ is the Cholesky factor ${\mathbf{L}}_{a}{\mathbf{L'}}_{a}=\mathbf{G}$; whereas the residual $\mathbf{e}$ was calculated as $\mathbf{e}={\mathbf{L}}_{e}{\mathbf{z}}_{e}$, where ${\mathbf{z}}_{e}\sim MVN({\mathbf{0}},\mathbf{I})$ and ${\mathbf{L}}_{e}$ is the Cholesky factor ${\mathbf{L}}_{e}{\mathbf{L'}}_{e}=\mathbf{R}$. The heritabilities for trait1 and trait2 were set to 0.1 and 0.5, respectively. As for the real PIC pig data, we defined two scenarios with 2314 and 578 animals, respectively. In order to simulate correlated traits with a genetic correlation of 0.3 and a residual correlation of 0.1, the variance components were set as follows:

$${\mathbf{G}}_{0}=\left[\begin{array}{cc}1.0& 0.67\\ 0.67& 5.0\end{array}\right],$$

$${\mathbf{R}}_{0}=\left[\begin{array}{cc}9.0& 0.67\\ 0.67& 5.0\end{array}\right].$$

In the statistical analysis, there are overall mean and no fixed effects. For each scenario, 10 replicates were simulated.

### Statistical model

Following Henderson and Quaas [1], the multi-trait mixed linear model for $n$ traits can be written as follows:

2 $${\mathbf{y}}_{i}={\mathbf{X}}_{i}{{\varvec{\upbeta}}}_{i}+{\mathbf{Z}}_{i}{\mathbf{a}}_{i}+{\mathbf{e}}_{i},\quad\boldsymbol{ }i={1,2}, \cdots ,n,$$

where ${\mathbf{y}}_{i}$ is the phenotype for trait $i$; ${{\varvec{\upbeta}}}_{i}$ is a vector of fixed effects associated with trait $i$; ${\mathbf{a}}_{i}$ is a vector of random additive genetic effects associated with trait $i$; ${\mathbf{e}}_{i}$ is a vector of residuals with trait $i$; and ${\mathbf{X}}_{i}$ and ${\mathbf{Z}}_{i}$ denote the incidence matrices relating the observations to the corresponding fixed and random effects. Let $\mathbf{y}={[{\mathbf{y'}}_{1},{\mathbf{y'}}_{2},\cdots ,{\mathbf{y'}}_{n}]'}$, ${\varvec{\upbeta}}={[{{\varvec{\upbeta'}}}_{1},{{\varvec{\upbeta'}}}_{2},\cdots ,{{\varvec{\upbeta'}}}_{n}]'}$, $\mathbf{a}={[{\mathbf{a'}}_{1},{\mathbf{a'}}_{2},\cdots ,{\mathbf{a'}}_{n}]'}$, and $\mathbf{e}={[{\mathbf{e'}}_{1},{\mathbf{e'}}_{2},\cdots ,{\mathbf{e'}}_{n}]'}$. Then, the mixed model equation for Model (1) can be expressed as follows:

3 $$\left[\begin{array}{cc}{\mathbf{X'}}{\mathbf{R}}^{-1}\mathbf{X}& {\mathbf{X'}}{\mathbf{R}}^{-1}\mathbf{Z}\\ {\mathbf{Z'}}{\mathbf{R}}^{-1}\mathbf{X}& {\mathbf{Z'}}{\mathbf{R}}^{-1}\mathbf{Z}+{\mathbf{G}}^{-1}\end{array}\right]\left[\begin{array}{c}\widehat{{\varvec{\upbeta}}}\\ \widehat{\mathbf{a}}\end{array}\right]=\left[\begin{array}{c}{\mathbf{X'}}{\mathbf{R}}^{-1}\mathbf{y}\\ {\mathbf{Z'}}{\mathbf{R}}^{-1}\mathbf{y}\end{array}\right],$$

where $\mathbf{G}$ and $\mathbf{R}$ are the covariance matrices associated with $\mathbf{a}$ and $\mathbf{e}$, respectively. Matrix $\mathbf{R}$ requires that each animal has either one record for all traits or none at all as is the case in our data.

### Estimation of variance components by REML and GS

Variance components were estimated using REML and GS with the airemlf90 and gibbs2f90 software (available at http://nce.ads.uga.edu/wiki/), respectively [27]. For REML, first we ran expectation maximization (EM)-REML for all the initial 10 iterations and then switched to average information (AI) in the final iteration because the EM algorithm is much more stable than the AI algorithm and is very robust to poor initial estimates and can thus provide a good starting point for the AI algorithm [28]. Convergence was assumed when changes in the ratios of the corresponding estimates between two consecutive rounds were less than ${10}^{-6}$. The asymptotic standard error (SE) was computed following Houle and Meyer [29], as implemented in airemllf90.

For GS, the conditional distribution of $\mathbf{y}$, given that the parameters are assumed to follow a multivariate normal distribution, is as follows:

4 $$\mathbf{y}|\mathbf{a},{\mathbf{R}}_{0} \sim MVN\left(\mathbf{Z}\mathbf{a},{\mathbf{R}}_{0}\bf{\otimes}\mathbf{I}\right).$$

In this study, the fixed effect was not included because the phenotypes were already corrected for environmental factors as described below. The additive genetic effects ($\mathbf{a}$) were assigned multivariate normal distributions with a mean vector of zeros:

5 $$\mathbf{a}|{\mathbf{G}}_{0},\mathbf{A} \sim MVN\left({\mathbf{0}},{\mathbf{G}}_{0}\bf{\otimes}\mathbf{A}\right),$$

and the residuals ($\mathbf{e}$) were assumed to follow:

6 $$\mathbf{e}|{\mathbf{R}}_{0} \sim MVN\left({\mathbf{0}},{\mathbf{R}}_{0}\bf{\otimes}\mathbf{I}\right).$$

For the covariance matrices (${\mathbf{G}}_{0}$ and ${\mathbf{R}}_{0}$), priors were derived from the IW distribution:

7 $$p\left({\mathbf{G}}_{0}|{v}_{\text{A}},{\mathbf{S}}_{\text{A}}\right)\propto {\left|{\mathbf{G}}_{0}\right|}^{-\frac{1}{2}\left({v}_{\text{A}}+n+1\right)}\text{exp}\left\{-\frac{1}{2}tr\left({\mathbf{G}}_{0}^{-1}{\mathbf{S}}_{\text{A}}^{-1}\right)\right\},$$

and

8 $$p\left({\mathbf{R}}_{0}|{v}_{\text{E}},{\mathbf{S}}_{\text{E}}\right)\propto {\left|{\mathbf{R}}_{0}\right|}^{-\frac{1}{2}\left({v}_{\text{E}}+n+1\right)}\text{exp}\left\{-\frac{1}{2}tr\left({\mathbf{R}}_{0}^{-1}{\mathbf{S}}_{\text{E}}^{-1}\right)\right\},$$

where ${\mathbf{S}}_{\text{A}}$ and ${\mathbf{S}}_{\text{E}}$ are the $n\times n$ scale parameter matrices, and ${v}_{\text{A}}$ and ${v}_{\text{E}}$ are the degrees of freedom for the additive genetic and residual covariances, respectively. The IW prior has gained popularity as the conjugate prior for multivariate normal distributions, facilitating computations via GS. In this study, we set ${\mathbf{S}}_{\text{A}}=\mathbf{I}$, ${\mathbf{S}}_{\text{E}}=\mathbf{I}$, ${v}_{\text{A}}=n$ and ${v}_{\text{E}}=n$. The posterior distribution for each parameter was obtained by integration of multivariate density functions, considering a single chain with 10,000 iterations. The first 1000 iterations were discarded as burn-in and the thinning interval of the chain was 10. Posterior mean and posterior standard deviation were calculated as the parameter estimates and their SE.

### Estimation of variance components by NUTS

In the Stan software, a Bayesian model is implemented by defining its likelihood and priors. Stan is an open-source software, with a publicly available manual online (https://mc-stan.org/users/documentation/). For the NUTS approach, we used RStan, which is the R interface for Stan.

We used a Lewandowski–Kurowicka–Joe (LKJ) distribution as a prior of the correlation. Following the separation strategy of Barnard et al. [30], the covariance matrices (${\mathbf{G}}_{0}$ and ${\mathbf{R}}_{0}$) were decomposed as ${\mathbf{G}}_{0}={{\varvec{\Lambda}}}_{\text{A}}{{\varvec{\Omega}}}_{\text{A}}{{\varvec{\Lambda}}}_{\text{A}}$ and ${\mathbf{R}}_{0}={{\varvec{\Lambda}}}_{\text{E}}{{\varvec{\Omega}}}_{\text{E}}{{\varvec{\Lambda}}}_{\text{E}}$, where ${{\varvec{\Lambda}}}_{\text{A}}$ and ${{\varvec{\Lambda}}}_{\text{E}}$ are the $n\times n$ diagonal matrices with the genetic and residual standard deviations, and ${{\varvec{\Omega}}}_{\text{A}}$ and ${{\varvec{\Omega}}}_{\text{E}}$ are the $n\times n$ genetic and residual correlation matrices, respectively. For the correlation matrices (${{\varvec{\Omega}}}_{\text{A}}$ and ${{\varvec{\Omega}}}_{\text{E}}$), priors were derived from the LKJ distribution with one positive scalar shape parameter $\eta$ [31]: ${{\varvec{\Omega}}}_{\text{A}}\sim LKJ(\eta )$ and ${{\varvec{\Omega}}}_{\text{E}}\sim LKJ(\eta )$. Here, we set the shape parameter for the genetic correlation as equal to that for the residual correlation. The posterior density function of the LKJ distribution for ${{\varvec{\Omega}}}_{\text{A}}$ is:

9 $$p\left({{\varvec{\Omega}}}_{\text{A}}|\eta \right)=\left[2\sum_{i=1}^{n-1}\left\{2\left(\eta -1\right)+n-i\right\}(n-i)\prod_{i=1}^{n-1}{\left\{Beta\left(\eta +\frac{\left(n-i-1\right)}{2},\eta +\frac{\left(n-i-1\right)}{2}\right)\right\}}^{n-i}\right]{|{{\varvec{\Omega}}}_{\text{A}}|}^{\eta -1},$$

and is proportional to the determinant of the correlation matrix raised to the $\eta -1$ power: $p\left({{\varvec{\Omega}}}_{\text{A}}|\eta \right)\propto {|{{\varvec{\Omega}}}_{\text{A}}|}^{\eta -1}$. Thus, the shape parameter $\eta$ tunes the strength of the correlations; $\eta =1$ leads to a uniform distribution on correlation matrices, while the magnitude of the correlations between components decreases as $\eta \to \infty$. In contrast, $0<\eta <1$ leads to low correlations. In the current study, the value of $\eta$ was set to 1 as the base value. Moreover, we investigated the effect of the scalar shape parameter $\eta$ of the LKJ distribution. In scenario 1, the values of $\eta$ were set to 0.25, 0.5, 1.0, 2.0 and 4.0.

For efficient calculation, we used Cholesky factor parameters for variance components. Let ${\mathbf{L}}_{\text{A}}$ be the Cholesky factor of $\mathbf{A}$: $\mathbf{A}={\mathbf{L}}_{\text{A}}{\mathbf{L'}}_{\text{A}}$. Let ${\mathbf{L}}_{{\Omega }_{\text{A}}}$ and ${\mathbf{L}}_{{\Omega }_{\text{E}}}$ be the Cholesky factors of ${{\varvec{\Omega}}}_{\text{A}}$ and ${{\varvec{\Omega}}}_{\text{E}}$: ${{\varvec{\Omega}}}_{\text{A}}={\mathbf{L}}_{{\Omega }_{\text{A}}}{\mathbf{L'}}_{{\Omega}_{\text{A}}}$ and ${{\varvec{\Omega}}}_{\text{E}}={\mathbf{L}}_{{{\varvec{\Omega}}}_{\text{E}}}{\mathbf{L'}}_{{{\varvec{\Omega}}}_{\text{E}}}$. Thus, the covariance matrices were redefined as: ${\mathbf{G}}_{0}={{\varvec{\Lambda}}}_{\text{A}}{\mathbf{L}}_{{\Omega }_{\text{A}}}{\mathbf{L'}}_{{\Omega }_{\text{A}}}{{\varvec{\Lambda}}}_{\text{A}}$ and ${\mathbf{R}}_{0}={{\varvec{\Lambda}}}_{\text{E}}{\mathbf{L}}_{{{\varvec{\Omega}}}_{\text{E}}}{\mathbf{L'}}_{{{\varvec{\Omega}}}_{\text{E}}}{{\varvec{\Lambda}}}_{\text{E}}$. Stan provides an implicit parameterization of the LKJ correlation matrix density in terms of its Cholesky factor. For the Cholesky factors ${\mathbf{L}}_{{\Omega }_{\text{A}}}$ and ${\mathbf{L}}_{{{\varvec{\Omega}}}_{\text{E}}}$ derived from the LKJ Cholesky distribution: ${\mathbf{L}}_{{\Omega }_{\text{A}}} \sim LKJCholesky(\eta )$ and ${\mathbf{L}}_{{{\varvec{\Omega}}}_{\text{E}}} \sim LKJCholesky\left(\eta \right).$ For example, ${\mathbf{L}}_{{\Omega }_{\text{A}}} \sim LKJCholesky(\eta )$ implies ${\mathbf{L}}_{{\Omega }_{\text{A}}}{\mathbf{L'}}_{{\Omega }_{\text{A}}} \sim LKJ(\eta )$. The priors of the diagonals of the genetic and residual standard deviations were assigned Cauchy distributions: ${{\varvec{\Lambda}}}_{\text{A}} \sim Cauchy(0, 5)$ and ${{\varvec{\Lambda}}}_{\text{E}} \sim Cauchy(0, 5)$. Hence, the random additive effects $\mathbf{a}$ was reshaped as $\mathbf{a}={\mathbf{L}}_{\text{A}}{\mathbf{z}}_{a}{\left({{\varvec{\Lambda}}}_{\text{A}}{\mathbf{L'}}_{{\Omega }_{\text{A}}}\right)}$. When ${\mathbf{z}}_{a}\sim MVN({\mathbf{0}},\mathbf{I})$:

10 $$\mathbf{a}|{\mathbf{L}}_{\text{A}},{{\varvec{\Lambda}}}_{\text{A}},{\mathbf{L}}_{{\Omega }_{\text{A}}}, {\mathbf{z}}_{a}\sim MVN\left({\mathbf{0}},{\mathbf{G}}_{0}\bf{\otimes}\mathbf{A}\right).$$

Stan provides an implicit parameterization of the multivariate normal density in terms of its Cholesky factor. The conditional distribution of $\mathbf{y}$ follows a multivariate normal Cholesky distribution:

11 $$\mathbf{y}|{\mathbf{L}}_{\text{A}},{{\varvec{\Lambda}}}_{\text{A}},{{\varvec{\Lambda}}}_{\text{E}},{\mathbf{L}}_{{\Omega }_{\text{A}}}, {\mathbf{L}}_{{{\varvec{\Omega}}}_{\text{E}}},{\mathbf{z}}_{a} \sim MVNCholesky\left(\mathbf{Z}\mathbf{a},{{\varvec{\Lambda}}}_{\text{E}}{\mathbf{L}}_{{{\varvec{\Omega}}}_{\text{E}}}\right),$$

which implies that $\mathbf{y}|{\mathbf{L}}_{\text{A}},{{\varvec{\Lambda}}}_{\text{A}},{{\varvec{\Lambda}}}_{\text{E}},{\mathbf{L}}_{{\Omega }_{\text{A}}}, {\mathbf{L}}_{{{\varvec{\Omega}}}_{\text{E}}},{\mathbf{z}}_{a} \sim MVN(\mathbf{Z}\mathbf{a},{\mathbf{R}}_{0}\bf{\otimes}\mathbf{I})$. The RStan code for NUTS with an LKJ prior in RStan is described in Additional file 1. In addition, we used the IW prior for NUTS to investigate whether either the sampling method or the prior, or both, contribute to the performance for the estimation of variance components. The RStan code for NUTS with the IW prior in RStan is described in Additional file 2. For NUTS, 2000 iterations were simulated to obtain posterior distributions and the first 1000 iterations were discarded as the warm-up phase.

Because for all but the most trivial model cases there is no analytical solution, NUTS uses a process called the leapfrog integration to draw a sketch of the posterior probability surface. The failures in this integrator are identified by “divergent transitions”, which basically means that the sampler is no longer following the surface of the model correctly [32]. To check for the presence of divergent transitions, after the warm-up phase, we investigated the two important parameters that affect divergent transitions: the number of steps and the tree depth. In Stan, the limits for number of steps and tree depth were set to 1000 and 10, respectively.

### Criteria for comparing methods

To investigate the accuracy of the estimation of variance components using the simulated data, we calculated two indices: the root mean square error (RMSE) and the mean absolute error (MAE). These indices of the estimator $\widehat{\theta }$ were calculated as follows:

12 $$\text{RMSE}\left(\theta \right)=\sqrt{\sum_{i=1}^{q}\frac{1}{q}{\left({\widehat{\theta }}_{i}-{\theta }_{i}\right)}^{2}},$$

13 $$\text{MAE}\left(\theta \right)=\sum_{i=1}^{q}\frac{1}{q}\left|\left({\widehat{\theta }}_{i}-{\theta }_{i}\right)\right|.$$

where $\widehat{\theta }$ is the estimated variance component obtained in each replication, $\theta$ is the true value used for the simulation and $q$ is the number of replicates. To avoid the differences of scales between scenarios, the relative RMSE and MAE were set to 1.0 for NUTS. Therefore, the relative RMSE and MAE were calculated by dividing the RMSE and MAE values by those for NUTS.

### Accuracy of estimated breeding values

The simulated population was divided into a training and a test population to investigate the accuracy of estimated breeding values. The training and test populations consisted of 2000 and 314 animals in Scenario 1 and of 500 and 78 animals in Scenario 2, respectively. The test population was randomly selected from the last generation. The training population had both phenotypic and genotypic values whereas the test population had only genotypic values. The accuracies of estimated breeding values were calculated from the Pearson’s correlations between the true and the estimated breeding values in the test population. In addition, we investigated the RMSE of estimated breeding values in the test population.

### Convergence diagnostics for MCMC

Establishing convergence of MCMC is one of the most important steps of Bayesian analysis. We used two MCMC diagnostic tools: the Gelman and Rubin’s convergence diagnostic [33] and the Geweke’s convergence diagnostic [34], which rely on multiple chains starting at initial points that are drawn from a density that is over-dispersed with respect to the target density. Using parallel chains, the convergence diagnostic ($\widehat{R}$) is calculated by comparing the within- and between-chain variances. A value of $\widehat{R}$ that is much higher than 1 indicates a lack of convergence. A cutoff value of 1.1 is generally used by MCMC practitioners, as recommended by Gelman et al. [35]. In this study, values of $\widehat{R}$ were calculated from three parallel chains. Geweke’s convergence diagnostic is based on a test for equality of the means of the first and last parts of a Markov chain. The test statistic is a standard z-score, which is calculated under the assumption that the two parts of the chain are asymptotically independent. The absolute value of the z-score exceeding 1.96 (5% cutoff point of the standard normal distribution) indicates a lack of convergence. We calculated the z-scores using the first 10% and the last 50% as two parts of the Markov chain. The two convergence diagnostic statistics were calculated using the R “coda” package [36].

## Results

### Comparison of parameter estimates

**Fig. 1:**
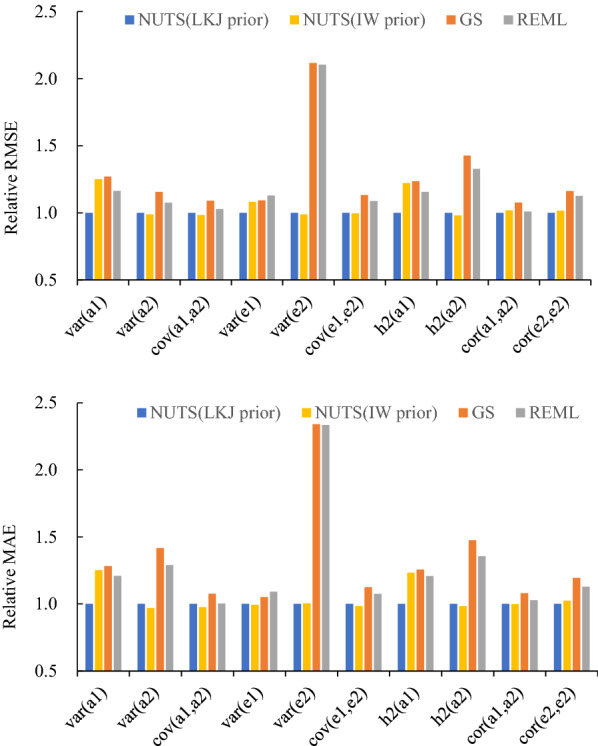
Plots of the relative root mean square error (RMSE) and mean absolute error (MAE) for all estimates in Scenario 1 using the simulated data. a1 and a2 are the additive genetic effects of trait1 and trait2, respectively; and e1 and e2 are the residuals of trait1 and trait2, respectively. *var* variance, *cov* covariance, *h2* heritability, *cor* correlation

**Fig. 2:**
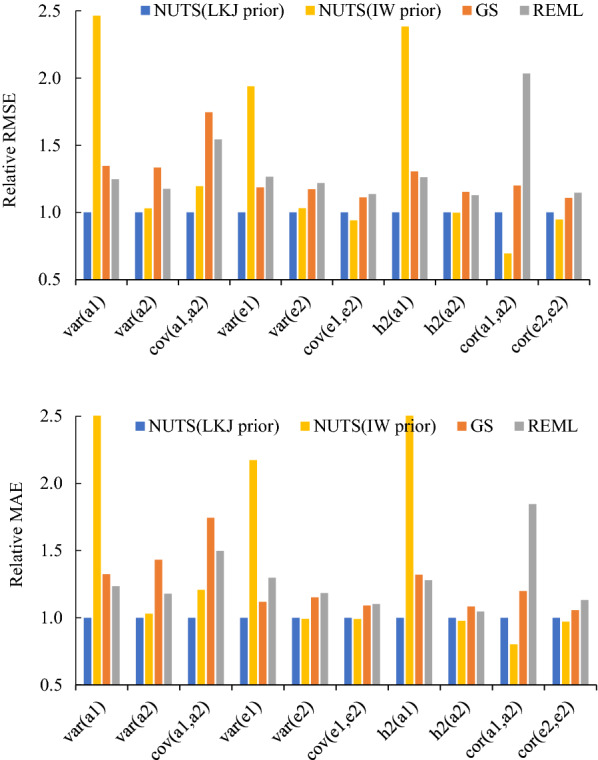
Plots of the relative root mean square error (RMSE) and mean absolute error (MAE) for all estimates in Scenario 2 using the simulated data. a1 and a2 are the additive genetic effects of trait1 and trait2, respectively; and e1 and e2 are the residuals of trait1 and trait2, respectively. *var* variance, *cov* covariance, *h2* heritability, *cor* correlation

**Fig. 3:**
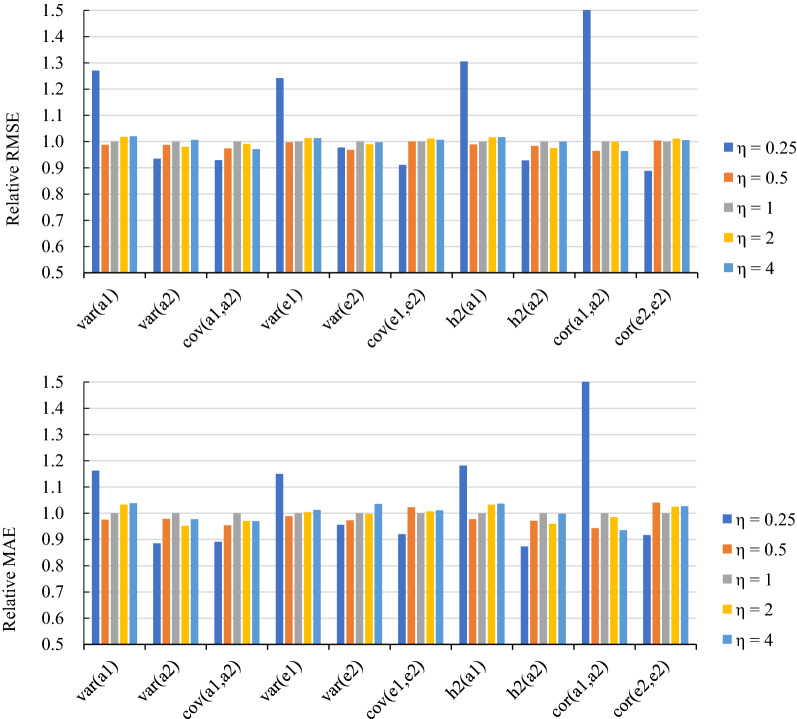
Plots of the relative root mean square error (RMSE) and mean absolute error (MAE) for all estimates using the No-U-Turn Sampler (NUTS) with different hyperparameters of LKJ distributions in Scenario 1 using the simulated data. a1 and a2 are the additive genetic effects of trait1 and trait2, respectively; and e1 and e2 are the residuals of trait1 and trait2, respectively. *var* variance, *cov* covariance, *h2* heritability, *cor* correlation

Compared to GS and REML, the average estimates of genetic variances and residual variances for trait2 obtained using NUTS with an LKJ prior were close to the true values in Scenario 1 (Table 1), but there was little difference between the estimates for all methods. The relative RMSE and MAE of the residual variances and heritabilities for trait2 were larger using GS and REML than those using NUTS with the LKJ and IW priors (Fig. 1). In Scenario 2, all the estimates obtained using NUTS with an LKJ prior were close to the true values whereas, in contrast to Scenario 1, the estimates with the other methods greatly differed from the true values (Table 2). For all the estimates, the relative RMSE and MAE using NUTS with an LKJ prior were smaller than those with GS and REML (Fig. 2). The relative RMSE and MAE of the estimates for trait1 using NUTS with an IW prior were quite large. The relative RMSE and MAE of some the parameters for the traits with a low heritability were high when $\eta$ = 0.25, whereas there were no differences in relative RMSE and MAE when $\eta \ge 0.5$ (Fig. 3).

**Table 1:** Estimated variance components, heritability and correlations in Scenario 1 using the simulated data

Parameter	True value	NUTS (LKJ prior)		NUTS (IW prior)		GS		REML	
		Mean	SE	Mean	SE	Mean	SE	Estimate	SE
Additive genetic (co)variances									
${\sigma }_{a}^{2}(trait1)$	1.05	1.11	0.26	1.29	0.21	1.24	0.30	1.19	0.27
${\sigma }_{a}^{2}(trait2)$	5.14	5.04	0.44	5.02	0.48	5.47	0.47	5.37	0.49
${\sigma }_{a}(trait1, trait2)$	0.70	0.64	0.24	0.61	0.31	0.64	0.26	0.65	0.27
Residual (co)variances									
${\sigma }_{e}^{2}(trait1)$	8.99	8.93	0.32	8.82	0.38	8.87	0.32	8.85	0.32
${\sigma }_{e}^{2}(trait2)$	5.03	4.96	0.22	4.96	0.26	4.70	0.24	4.70	0.24
${\sigma }_{e}(trait1, trait2)$	0.65	0.66	0.19	0.67	0.20	0.66	0.26	0.64	0.20
Heritabilities									
${h}^{2}(trait1)$	0.11	0.11	0.03	0.13	0.02	0.12	0.03	0.12	0.03
${h}^{2}(trait2)$	0.50	0.50	0.03	0.50	0.03	0.54	0.03	0.53	0.03
Additive genetic correlations									
${r}_{a}(trait1, trait2)$	0.30	0.26	0.10	0.24	0.10	0.24	0.10	0.26	0.11
Residual correlations									
${r}_{e}(trait1, trait2)$	0.10	0.10	0.03	0.10	0.03	0.10	0.03	0.10	0.03

*NUTS* No-U-Turn sampler, *LKJ* Lewandowski-Kurowicka-Joe, *IW* inverse-Wishart, *GS* Gibbs sampling, *REML* restricted maximum likelihood, *SE* standard error

${\sigma }_{a}^{2}$: additive genetic variance, ${\sigma }_{a}$: additive genetic covariance, ${\sigma }_{e}^{2}$: residual variance, ${\sigma }_{e}$: residual covariance, ${h}^{2}$: heritability, ${r}_{a}$: additive genetic correlation, ${r}_{e}$: residual correlation

**Table 2:** Estimated variance components, heritability and correlations in Scenario 2 using the simulated data

Parameter	True value	NUTS (LKJ prior)		NUTS (IW prior)		GS		REML	
		Mean	SE	Mean	SE	Mean	SE	Estimate	SE
Additive genetic (co)variances									
${\sigma }_{a}^{2}(trait1)$	0.98	1.00	0.36	1.79	0.20	1.29	0.35	1.06	0.44
${\sigma }_{a}^{2}(trait2)$	4.89	5.32	1.48	5.37	1.40	5.84	1.46	5.54	1.49
${\sigma }_{a}(trait1, trait2)$	0.59	0.57	0.29	0.72	0.35	0.86	0.46	0.75	0.47
Residual (co)variances									
${\sigma }_{e}^{2}(trait1)$	9.06	9.06	0.44	8.45	0.39	8.90	0.45	8.94	0.50
${\sigma }_{e}^{2}(trait2)$	5.02	4.82	0.59	4.78	0.59	4.61	0.63	4.60	0.66
${\sigma }_{e}(trait1, trait2)$	0.84	0.88	0.42	0.82	0.41	0.75	0.46	0.80	0.46
Heritabilities									
${h}^{2}(trait1)$	0.10	0.10	0.03	0.17	0.02	0.13	0.03	0.11	0.04
${h}^{2}(trait2)$	0.50	0.52	0.08	0.52	0.08	0.55	0.08	0.54	0.09
Additive genetic correlations									
${r}_{a}(trait1, trait2)$	0.27	0.31	0.20	0.24	0.12	0.34	0.21	0.40	0.33
Residual correlations									
${r}_{e}(trait1, trait2)$	0.12	0.13	0.06	0.13	0.07	0.12	0.07	0.13	0.07

*NUTS* No-U-Turn sampler, *LKJ* Lewandowski-Kurowicka-Joe, *IW* inverse-Wishart, *GS* Gibbs sampling, *REML* restricted maximum likelihood, *SE* standard error

${\sigma }_{a}^{2}$: additive genetic variance, ${\sigma }_{a}$: additive genetic covariance, ${\sigma }_{e}^{2}$: residual variance, ${\sigma }_{e}$: residual covariance, ${h}^{2}$: heritability, ${r}_{a}$: additive genetic correlation, ${r}_{e}$: residual correlation

**Fig. 4:**
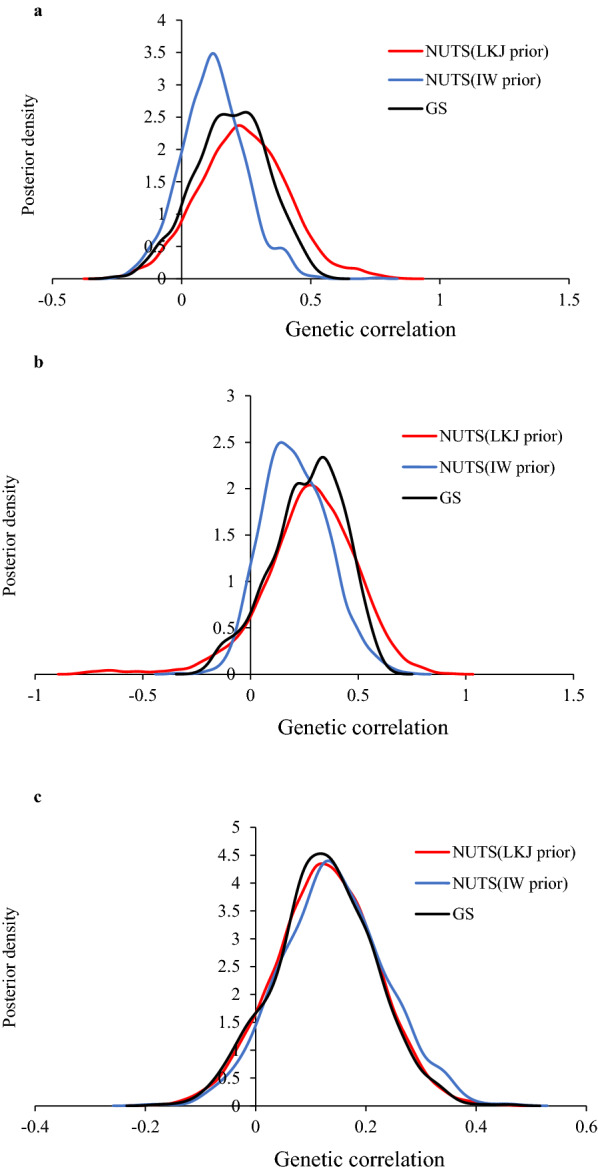
Posterior density plots for genetic correlations using the No-U-Turn Sampler (NUTS) with an LKJ prior (red line), NUTS with an IW prior (blue line) and the Gibbs sampler (GS) (black line) in Scenario 1 using the real PIC pig data. **a** T1 and T2; **b** T1 and T3; and **c** T2 and T3

The parameter estimates for the real PIC pig data obtained with the trivariate animal models using NUTS, GS and REML in Scenarios 1 and 2 are in Tables 3 and 4, respectively. In Scenario 1, the estimates of the genetic correlations between T1 and T2, and between T1 and T3, differed between the three methods whereas the other parameter estimates were almost the same. In Scenario 2, the differences in the estimates of genetic correlations were larger than those in Scenario 1. In particular, the estimates of the genetic correlation between T1 and T2 and their SE using GS and REML were quite high (Table 4). The estimates of the genetic variance and heritability using NUTS with an IW prior were higher than those with the other methods. The posterior distributions of GS for the genetic correlations between T1 and T2, and between T1 and T3, were skewed compared to those of NUTS (Fig. 4).

**Table 3:** Estimated variance components, heritability and correlations in Scenario 1 using the real PIC pig data

Parameter	NUTS (LKJ prior)		NUTS (IW prior)		GS		REML	
	Mean	SE	Mean	SE	Mean	SE	Estimate	SE
Additive (co)variances								
${\sigma }_{a}^{2}(T1)$	0.03	0.02	0.07	0.02	0.05	0.02	0.04	0.02
${\sigma }_{a}^{2}(T2)$	0.23	0.03	0.24	0.03	0.26	0.03	0.25	0.03
${\sigma }_{a}^{2}(T3)$	0.24	0.04	0.25	0.04	0.27	0.04	0.26	0.04
${\sigma }_{a}(T1,T2)$	0.02	0.01	0.02	0.05	0.02	0.02	0.02	0.02
${\sigma }_{a}(T1,T3)$	0.02	0.02	0.03	0.07	0.03	0.02	0.03	0.02
${\sigma }_{a}(T2,T3)$	0.03	0.02	0.03	0.02	0.03	0.02	0.03	0.02
Residual (co)variances								
${\sigma }_{e}^{2}(T1)$	0.97	0.03	0.94	0.03	0.96	0.03	0.96	0.03
${\sigma }_{e}^{2}(T2)$	0.62	0.02	0.62	0.02	0.61	0.02	0.61	0.02
${\sigma }_{e}^{2}(T3)$	0.75	0.03	0.75	0.03	0.73	0.03	0.73	0.03
${\sigma }_{e}(T1,T2)$	0.01	0.02	0.02	0.02	0.01	0.02	0.01	0.02
${\sigma }_{e}(T1,T3)$	0.00	0.04	0.00	0.04	0.00	0.03	0.00	0.02
${\sigma }_{e}(T2,T3)$	0.02	0.02	0.01	0.05	0.01	0.02	0.01	0.02
Heritabilities								
${h}^{2}(T1)$	0.03	0.02	0.07	0.02	0.05	0.02	0.04	0.02
${h}^{2}(T2)$	0.27	0.03	0.27	0.03	0.30	0.03	0.29	0.03
${h}^{2}(T3)$	0.24	0.03	0.25	0.03	0.27	0.03	0.26	0.03
Additive genetic correlations								
${r}_{a}(T1,T2)$	0.24	0.17	0.12	0.12	0.19	0.14	0.25	0.25
${r}_{a}(T1,T3)$	0.27	0.22	0.21	0.15	0.26	0.17	0.37	0.34
${r}_{a}(T2,T3)$	0.13	0.09	0.14	0.03	0.12	0.09	0.14	0.10
Residual genetic correlations								
${r}_{e}(T1,T2)$	0.02	0.02	0.02	0.03	0.02	0.03	0.02	0.03
${r}_{e}(T1,T3)$	0.00	0.04	0.00	0.04	0.00	0.05	0.00	0.03
${r}_{e}(T2,T3)$	0.02	0.03	0.02	0.03	0.02	0.03	0.02	0.03

*NUTS* No-U-Turn sampler, *LKJ* Lewandowski-Kurowicka-Joe, *IW* inverse-Wishart, *GS* Gibbs sampling, *REML* restricted maximum likelihood, *SE* standard error

${\sigma }_{a}^{2}$: additive genetic variance, ${\sigma }_{a}$: additive genetic covariance, ${\sigma }_{e}^{2}$: residual variance, ${\sigma }_{e}$: residual covariance, ${h}^{2}$: heritability, ${r}_{a}$: additive genetic correlation, ${r}_{e}$: residual correlation

**Table 4:** Estimated variance components, heritability and correlations in Scenario 2 using the real PIC pig data

Parameter	NUTS (LKJ prior)		NUTS (IW prior)		GS		REML	
	Mean	SE	Mean	SE	Mean	SE	Estimate	SE
Additive (co)variances								
${\sigma }_{a}^{2}(T1)$	0.02	0.01	0.13	0.04	0.01	0.01	0.02	0.01
${\sigma }_{a}^{2}(T2)$	0.34	0.07	0.34	0.07	0.38	0.08	0.28	0.06
${\sigma }_{a}^{2}(T3)$	0.28	0.08	0.30	0.08	0.35	0.09	0.31	0.09
${\sigma }_{a}(T1,T2)$	0.01	0.02	0.01	0.09	0.04	0.03	0.04	0.03
${\sigma }_{a}(T1,T3)$	0.01	0.02	0.04	0.04	0.02	0.04	0.06	0.04
${\sigma }_{a}(T2,T3)$	0.05	0.05	0.05	0.05	0.06	0.06	0.06	0.06
Residual (co)variances								
${\sigma }_{e}^{2}(T1)$	1.04	0.06	0.96	0.07	1.05	0.07	1.04	0.06
${\sigma }_{e}^{2}(T2)$	0.60	0.06	0.59	0.06	0.58	0.06	0.69	0.04
${\sigma }_{e}^{2}(T3)$	0.67	0.07	0.65	0.07	0.64	0.07	0.65	0.07
${\sigma }_{e}(T1,T2)$	0.04	0.04	0.04	0.05	0.02	0.04	0.02	0.05
${\sigma }_{e}(T1,T3)$	0.00	0.04	− 0.02	0.08	0.00	0.05	− 0.07	0.04
${\sigma }_{e}(T2,T3)$	− 0.06	0.05	-0.06	0.02	− 0.07	0.05	− 0.07	0.05
Heritabilities								
${h}^{2}(T1)$	0.02	0.02	0.12	0.04	0.01	0.01	0.01	0.01
${h}^{2}(T2)$	0.36	0.06	0.37	0.06	0.39	0.07	0.28	0.05
${h}^{2}(T3)$	0.29	0.07	0.32	0.08	0.35	0.08	0.32	0.08
Additive genetic correlations								
${r}_{a}(T1,T2)$	0.13	0.32	0.05	0.20	0.62	0.44	0.63	0.53
${r}_{a}(T1,T3)$	0.12	0.36	0.19	0.23	0.32	0.48	0.88	0.53
${r}_{a}(T2,T3)$	0.15	0.16	0.16	0.17	0.16	0.17	0.21	0.21
Residual genetic correlations								
${r}_{e}(T1,T2)$	0.05	0.05	0.05	0.06	0.02	0.06	0.02	0.05
${r}_{e}(T1,T3)$	0.00	0.05	− 0.02	0.11	− 0.02	0.09	− 0.09	0.05
${r}_{e}(T2,T3)$	− 0.09	0.06	− 0.10	0.04	− 0.11	0.08	− 0.12	0.08

*NUTS* No-U-Turn sampler, *LKJ* Lewandowski-Kurowicka-Joe, *IW* inverse-Wishart, *GS* Gibbs sampling, *REML* restricted maximum likelihood, *SE* standard error

${\sigma }_{a}^{2}$: additive genetic variance, ${\sigma }_{a}$: additive genetic covariance, ${\sigma }_{e}^{2}$: residual variance, ${\sigma }_{e}$: residual covariance, ${h}^{2}$: heritability, ${r}_{a}$: additive genetic correlation, ${r}_{e}$: residual correlation

There were no differences in accuracies and RMSE of estimated breeding values between the four methods in Scenario 1. In Scenario 2, the accuracies of estimated breeding values for trait1 using NUTS with the LKJ and IW priors were 14.8% and 11.1% higher than those using GS and REML, respectively (Table 5). The RMSE of trait2 using NUTS with the LKJ and IW priors were smaller than those using GS and REML.

**Table 5:** Accuracy (correlation coefficient) and root mean square error (RMSE) of estimated breeding value (and standard deviation) using the simulated data

		NUTS (LJK prior)	NUTS (IW prior)	GS	REML
Correlation coefficient	Scenario 1				
	Trait1	0.48 (0.08)	0.49 (0.08)	0.48 (0.08)	0.49 (0.08)
	Trait2	0.72 (0.06)	0.72 (0.06)	0.73 (0.06)	0.73 (0.06)
	Scenario 2				
	Trait1	0.27 (0.11)	0.27 (0.11)	0.23 (0.13)	0.24 (0.11)
	Trait2	0.51 (0.07)	0.50 (0.07)	0.45 (0.13)	0.50 (0.07)
RMSE	Scenario 1				
	Trait1	0.88 (0.05)	0.88 (0.05)	0.88 (0.05)	0.88 (0.05)
	Trait 2	1.55 (0.07)	1.57 (0.8)	1.55 (0.07)	1.55 (0.08)
	Scenario 2				
	Trait1	0.96 (0.10)	0.97 (0.12)	0.97 (0.07)	0.96 (0.08)
	Trait2	1.89 (0.20)	1.89 (0.22)	1.96 (0.27)	1.94 (0.30)

*NUTS* No-U-Turn sampler, *LKJ* Lewandowski-Kurowicka-Joe, *IW* inverse-Wishart, *GS* Gibbs sampling, *REML* restricted maximum likelihood

### Performances of MCMC sampling using NUTS and GS

The $\widehat{R}$ values of the Gelman and Rubin’s R convergence diagnostics and the z-scores of Geweke’s convergence diagnostics in Scenarios 1 and 2 using simulated and real PIC pig data are in Tables S1, S2, S3 and S4, respectively, (see Additional file 3: Table S1, Additional file 4: Table S2, Additional file 5: Table S3 and Additional file 6: Table S4). The convergences of the MCMC samplings using NUTS with the LKJ prior were established. Using NUTS with the IW prior, the $\widehat{R}$ values were smaller than the value of 1.1 set as criterion, but the z-scores for the five parameters in Scenario 2 using simulated data exceeded the criterion value (1.96). Using GS, some of the $\widehat{R}$ values and the z-scores exceeded the criterion values with both the simulated and real PIC pig data.

There were no divergent transitions in both the simulated data and real PIC pig data (see Additional file 7: Table S5). The numbers of leapfrog steps ranged from 36.7 to 63.7 and the tree depths ranged from 5.0 to 5.6. These two parameters were below the limit values defined in Stan.

### Computing time

Total computing times for REML were much shorter than those for NUTS and GS (See Additional file 8: Table S6). The computing times per MCMC iteration for NUTS were longer than those for GS in all cases. The total computing times of 2000 iterations for NUTS were similar to those of 10,000 iterations for GS in Scenario 1 for both the simulated and real PIC pig data. In Scenario 2, the total computing times for NUTS with the LKJ and IW priors were 2.5 times longer compared to those for GS.

## Discussion

Multi-trait analysis using mixed models tends to be more powerful and to provide more accurate estimates than single-trait analysis because the former method can take the underlying correlation structure that is present in multi-trait data into account. Thus, the estimation of (co)variance and correlation parameters in multi-trait analysis is an important topic in animal breeding programs. However, Bayesian and non-Bayesian inferences for multi-trait mixed models are complex. In this study, we focused on the NUTS approach and implemented NUTS with LKJ and IW priors for a multi-trait animal model using the recently developed software Stan, and compared the results with the commonly used REML and GS methods. The results obtained with the simulated and real pig data indicate that the estimation of genetic parameters for a multi-trait animal model is improved by using NUTS with an LKJ prior, particularly when the population size is small. Moreover, for real pig data, NUTS can provide the unimodal and bilaterally symmetrical posterior distributions of genetic correlations regardless of the level of the heritability.

The NUTS approach has two advantages over GS. First, the NUTS algorithm is extremely effective for the MCMC sampling process because it can generate samples from a wide range of parameter spaces with a high level of acceptance probability and automatic tuning of the hyperparameters of HMC. Nishio and Arakawa [22] demonstrated how to use Stan for a single-trait animal model and showed that the mixing properties of Stan were better than the GS with no tuning. Second, conjugate priors are not necessary for NUTS, which opens up the possibility of other potentially beneficial priors. The choice of prior is important and can influence the posterior, particularly when the amount of data is small. A common choice is a conjugate prior, where both the prior and the posterior have the same distributional form. Typically, a conjugate prior is chosen to provide analytical solutions for the posterior and is a requirement for GS. The natural conjugate prior for a multivariate normal distribution is the IW distribution [28]. However, IW priors impose a degree of informativity and the posterior inferences are sensitive to the choice of hyperparameters [37] and there is an a priori dependence between correlations and variances [38]. These characteristics of the prior frequently result in biased estimates in the analysis of small datasets. In this study, we used the LKJ distributions as priors of correlations in the NUTS approach. This is one of the separation strategies in which the standard deviations and correlations are modeled independently and then combined to form a prior on the covariance matrix [30]. Alvarez et al. [39] showed that the separation strategy resulted in a better inference property than the use of an IW prior. In this study, we compared the performance of NUTS with LKJ and IW priors. When the population size was small, the RMSE and MAE of trait1 (trait with a low heritability) using NUTS with an IW prior were notably larger than those using NUTS with an LKJ prior. In addition, Geweke’s convergence diagnostic (z-scores) using NUTS with an IW prior were larger than the criterion value. These results indicate that the performance of NUTS with an LKJ prior was superior to that of NUTS with an IW prior for estimating variance components and MCMC sampling convergence.

For an LKJ distribution, one positive scalar hyperparameter ($\eta$) tunes the strength of the correlations. In this study, we varied the values of $\eta$ from 0.25 to 4.0 in the simulation Scenario 1. The effect of $\eta$ on the performance of NUTS with an LKJ prior was negligible except when the values of $\eta$ were very small. Our results indicate that values of $\eta$ exceeding 0.5 are preferable. The Stan manual also recommends $\eta \ge 1$.

Few studies have compared the performance of REML and GS for the estimation of variance components in a multi-trait analysis. In the animal breeding literature, Van Tassel and Van Vleck [19] reported that the posterior means of GS and REML estimates for additive genetic variances and correlations were quite similar for traits with a high heritability. In the plant breeding literature, Waldmann and Ericsson [40] reported that REML estimates were accurate and that the posterior means of GS were overestimated based on the results of two simulated traits with heritabilities of 0.1 and 0.5, respectively. These results are in concordance with those of our study: the estimates of the additive genetic variances and heritabilities obtained with GS were overestimated when using simulated data, particularly when the population size was small. However, in the simulation study of Mathew et al. [41], GS provided better estimates for the additive genetic correlations than the REML approach with a dataset for traits with a low heritability. The performance of GS could be strongly influenced by a prior with a low heritability. Thus, the choices of inference methods and priors are complex for multi-trait analyses and could be solved by using the NUTS algorithm with an LKJ prior as shown here.

The predictions of breeding values by NUTS were superior to those by GS and REML when the population size was small. In animal breeding, there are cases where small datasets need to be analyzed, e.g. for rare breeds that are maintained in small population sizes. In Japan, 18 local public animal experimental stations have performed selection experiments in closed small populations for several generations using estimated breeding values. Moreover, for many difficult-to-measure or expensive traits, such as methane emission, heat tolerance, individual feed intake or immune response, NUTS is a promising sampling method for multi-trait analysis.

In this study, we used genomic information to generate the relationship matrix. The MCMC implementation of the animal model can become extremely slow when using a genomic relationship matrix instead of a pedigree-based relationship matrix. In order to decrease computing requirements, Villemereuil [42] suggested two promising approaches: Integrated Nested Laplace Approximations (INLA) [43] and HMC. Mathew et al. [41] showed that the genetic parameter estimates for the INLA approach and the MCMC method were almost the same in a multi-trait animal model when relationship matrices were dense. They concluded that the INLA approach could be a fast alternative to MCMC methods for multi-trait animal models. Our study showed the computing times of NUTS derived from HMC. The total and per iteration computing times of NUTS were longer than those of GS with both the simulated and real PIC pig data when the sample size was large. Conversely, the convergence performance of NUTS with an LKJ prior was superior to that of GS because the convergence conditions were sufficiently established for both the Gelman and Rubin’s R convergence and the Geweke’s convergence diagnostics in all scenarios. In addition, the effective sample size for NUTS is much larger than that for GS [22]. These results indicate that the computing time of NUTS with an LKJ prior could be reduced by decreasing the number of MCMC iterations. Recently, Arakawa et al. [23] developed the HMC method with optimized tunings of hyperparameters in a single-trait animal model. This method outperformed GS in terms of sampling from a wider range of parameter spaces. The computing time for their method was similar to that for GS. Thus, further study is needed to apply this method to multi-trait animal models.

Developing a program for NUTS is challenging because of its very complex algorithm; however, this can be overcome by using Stan, which has a simple programming language. In this study, we used Stan because a Bayesian model is implemented by defining its likelihood and priors. Recently, Burkner [44] developed the “brms” package, which allows R users to easily specify a wide range of Bayesian single- and multi-level models that are fitted with Stan. This package allows the writing of models in a relatively straightforward R syntax. Thus, it might be possible to write the program code of a multi-trait animal model easily using brms.

## Conclusions

In this paper, we applied the NUTS approach with LKJ and IW priors to a multi-trait animal model and showed its performance for estimating variance components and breeding values. The simulated data showed that, compared to NUTS with an IW prior, GS and REML, most of the estimates of genetic parameters obtained by using NUTS with an LKJ prior were closer to the true values and RMSE and MAE were smaller. These tendencies were remarkable when the population size was small. The convergence performances of MCMC samplings using NUTS with an LKJ prior were superior to those of NUTS with an IW prior and to GS. Moreover, the accuracies of estimated breeding values for NUTS with LKJ and IW priors were higher than those for GS and REML when the population size was small. The real PIC pig data showed that the effect of population size on estimating genetic correlations using NUTS with an LKJ prior was smaller than that using GS and REML. For both the simulated and real PIC pig data, the genetic variances and heritabilities using NUTS with an IW prior were overestimated for traits with a low heritability when the population size was small. Developing a NUTS program for a multi-trait animal model is challenging because of its very complex algorithm but this can be overcome by using Stan and its simple programming language. However, application of NUTS to large datasets requires further study because the NUTS algorithm requires much computing time. Therefore, we conclude that NUTS with an LKJ prior could be an alternative sampling method for multi-trait analysis in animal breeding, particularly when the population size is small.

## Supplementary Information

**Additional file 1**. RStan code for the NUTS algorithm with an LKJ prior of a multi-trait animal model. RStan is the R interface to Stan. The user writes the analysis model in the test.stan file and runs Stan by using test.stan in R. The user needs to input the following parameters: J, number of fixed effects; K, number of traits; Q, total number of animals; N, number of observations; X, design matrix for fixed effects; Z, design matrix for random effects; Y, response variable; and A, relationship matrix.

**Additional file 2**. RStan code for the NUTS algorithm with an IW prior of a multi-trait animal model.

**Additional file 3: Table S1**. Gelman and Rubin’s R convergence diagnostic and Geweke’s convergence diagnostic in Scenario 1 for the simulated data.

**Additional file 4: Table S2**. Gelman and Rubin’s R convergence diagnostic and Geweke’s convergence diagnostic in Scenario 2 for the simulated data.

**Additional file 5: Table S3**. Gelman and Rubin’s R convergence diagnostic and Geweke’s convergence diagnostic in Scenario 1 for the PIC pig data.

**Additional file 6: Table S4**. Gelman and Rubin’s R convergence diagnostic and Geweke’s convergence diagnostic in Scenario 2 for the PIC pig data.

**Additional file 7: Table S5**. Presence of divergence and parameters that affect divergence for the No-U-Turn Sampler.

**Additional file 8: Table S6**. Number of iterations and computing time (seconds) for estimating variance components.

## Data Availability

The R codes to implement the NUTS algorithm in Stan are written in Additional files 1 and 2.
