# Supplementary material for: Performance of the No-U-Turn sampler in multi-trait variance component estimation using genomic data
Source: Genet Sel Evol. 2022 Jul 11;54:51. doi: 10.1186/s12711-022-00743-5 (PMC9275044; doi:10.1186/s12711-022-00743-5)
Supplement: Supplementary file 3 — Additional file 3: Table S1. Gelman and Rubin’s R convergence diagnostic and Geweke’s convergence diagnostic in Scenario 1 for the simulated data. [file 12711_2022_743_MOESM3_ESM.docx]

**Table S1** Gelman and Rubin’s R convergence diagnostic and Geweke’s convergence diagnostic in scenario 1 of simulated data

| Parameter | Gelman and Rubin’s R convergence diagnostic ($\hat{R}$) | | | Geweke’s convergence diagnostic (z-score) | | |
| --- | --- | --- | --- | --- | --- | --- |
|  | NUTS (LKJ prior) | NUTS (IW prior) | GS | NUTS (LKJ prior) | NUTS (IW prior) | GS |
| Additive genetic (co)variances |  |  |  |  |  |  |
| $\sigma_{a}^{2}(trait1)$ | 1.00 | 1.00 | 1.03 | 0.98 | 0.64 | 0.54 |
| $\sigma_{a}^{2}(trait2)$ | 1.00 | 1.01 | 1.00 | 0.88 | 0.85 | 0.86 |
| $\sigma_{a}(trait1, trait2)$ | 1.01 | 1.02 | 1.00 | 0.96 | 0.73 | 0.51 |
| Residual (co)variances |  |  |  |  |  |  |
| $\sigma_{e}^{2}(trait1)$ | 1.00 | 1.00 | 1.00 | 0.51 | 0.78 | 0.51 |
| $\sigma_{e}^{2}(trait2)$ | 1.00 | 1.01 | 1.00 | 1.25 | 1.34 | 0.81 |
| $\sigma_{e}(trait1, trait2)$ | 1.01 | 1.01 | 1.00 | 1.51 | 0.57 | 0.44 |
| Heritabilities |  |  |  |  |  |  |
| $h^{2}(trait1)$ | 1.00 | 1.01 | 1.02 | 0.85 | 0.69 | 0.50 |
| $h^{2}(trait2)$ | 1.00 | 1.01 | 1.00 | 1.08 | 0.97 | 0.85 |
| Additive genetic correlations |  |  |  |  |  |  |
| $r_{a}(trait1, trait2)$ | 1.01 | 1.01 | 1.01 | 0.97 | 0.86 | 0.24 |
| Residual correlations |  |  |  |  |  |  |
| $r_{e}(trait1, trait2)$ | 1.01 | 1.01 | 1.00 | 1.52 | 0.57 | 0.46 |
