# Supplementary material for: Performance of the No-U-Turn sampler in multi-trait variance component estimation using genomic data
Source: Genet Sel Evol. 2022 Jul 11;54:51. doi: 10.1186/s12711-022-00743-5 (PMC9275044; doi:10.1186/s12711-022-00743-5)
Supplement: Supplementary file 4 — Additional file 4: Table S2. Gelman and Rubin’s R convergence diagnostic and Geweke’s convergence diagnostic in Scenario 2 for the simulated data. [file 12711_2022_743_MOESM4_ESM.docx]

**Table S2** Gelman and Rubin’s R convergence diagnostic and Geweke’s convergence diagnostic in scenario 2 of simulated data

| Parameter | Gelman and Rubin’s R convergence diagnostic ($\hat{R}$) | | | Geweke’s convergence diagnostic (z-score) | | |
| --- | --- | --- | --- | --- | --- | --- |
|  | NUTS (LKJ prior) | NUTS (IW prior) | GS | NUTS (LKJ prior) | NUTS (IW prior) | GS |
| Additive genetic (co)variances |  |  |  |  |  |  |
| $\sigma_{a}^{2}(trait1)$ | 1.02 | 1.00 | 1.02 | 0.90 | 2.08 | 1.77 |
| $\sigma_{a}^{2}(trait2)$ | 1.00 | 1.01 | 1.01 | 0.70 | 1.07 | 0.45 |
| $\sigma_{a}(trait1, trait2)$ | 1.02 | 1.05 | 1.02 | 1.49 | 1.52 | 1.91 |
| Residual (co)variances |  |  |  |  |  |  |
| $\sigma_{e}^{2}(trait1)$ | 1.02 | 1.00 | 1.00 | 1.28 | 2.19 | 1.64 |
| $\sigma_{e}^{2}(trait2)$ | 1.00 | 1.01 | 1.00 | 0.64 | 1.21 | 0.77 |
| $\sigma_{e}(trait1, trait2)$ | 1.01 | 1.02 | 1.01 | 1.42 | 1.99 | 1.49 |
| Heritabilities |  |  |  |  |  |  |
| $h^{2}(trait1)$ | 1.02 | 1.00 | 1.02 | 0.95 | 2.16 | 1.77 |
| $h^{2}(trait2)$ | 1.00 | 1.01 | 1.01 | 0.67 | 1.10 | 0.64 |
| Additive genetic correlations |  |  |  |  |  |  |
| $r_{a}(trait1, trait2)$ | 1.05 | 1.04 | 1.02 | 1.32 | 1.66 | 2.24 |
| Residual correlations |  |  |  |  |  |  |
| $r_{e}(trait1, trait2)$ | 1.01 | 1.02 | 1.01 | 1.39 | 2.03 | 1.49 |
