# Supplementary material for: Performance of the No-U-Turn sampler in multi-trait variance component estimation using genomic data
Source: Genet Sel Evol. 2022 Jul 11;54:51. doi: 10.1186/s12711-022-00743-5 (PMC9275044; doi:10.1186/s12711-022-00743-5)
Supplement: Supplementary file 5 — Additional file 5: Table S3. Gelman and Rubin’s R convergence diagnostic and Geweke’s convergence diagnostic in Scenario 1 for the PIC pig data. [file 12711_2022_743_MOESM5_ESM.docx]

**Table S3** Gelman and Rubin’s R convergence diagnostic and Geweke’s convergence diagnostic in scenario 1 of PIC pig data

| Parameter | Gelman and Rubin’s R convergence diagnostic ($\hat{R}$) | | | Geweke’s convergence diagnostic (z-score) | | |
| --- | --- | --- | --- | --- | --- | --- |
|  | NUTS (LKJ prior) | NUTS (IW prior) | GS | NUTS (LKJ prior) | NUTS (IW prior) | GS |
| Additive (co)variances |  |  |  |  |  |  |
| $\sigma_{a}^{2}(T1)$ | 1.01 | 1.01 | 1.60 | 1.58 | 1.53 | 1.95 |
| $\sigma_{a}^{2}(T2)$ | 1.00 | 1.00 | 1.00 | 0.62 | 1.05 | 0.93 |
| $\sigma_{a}^{2}(T3)$ | 1.00 | 1.01 | 1.01 | 1.25 | 1.27 | 0.62 |
| $\sigma_{a}(T1,T2)$ | 1.01 | 1.00 | 1.14 | 0.40 | 1.59 | 1.51 |
| $\sigma_{a}(T1,T3)$ | 1.01 | 1.03 | 1.14 | 1.13 | 1.04 | 1.38 |
| $\sigma_{a}(T2,T3)$ | 1.00 | 1.01 | 1.01 | 0.81 | 0.68 | 1.30 |
| Residual (co)variances |  |  |  |  |  |  |
| $\sigma_{e}^{2}(T1)$ | 1.00 | 1.00 | 1.08 | 1.49 | 0.52 | 2.11 |
| $\sigma_{e}^{2}(T2)$ | 1.00 | 1.00 | 1.00 | 0.70 | 1.15 | 1.30 |
| $\sigma_{e}^{2}(T3)$ | 1.00 | 1.01 | 1.00 | 1.27 | 1.67 | 0.52 |
| $\sigma_{e}(T1,T2)$ | 1.00 | 1.00 | 1.01 | 0.87 | 1.55 | 1.70 |
| $\sigma_{e}(T1,T3)$ | 1.00 | 1.01 | 1.01 | 1.05 | 1.16 | 1.52 |
| $\sigma_{e}(T2,T3)$ | 1.00 | 1.00 | 1.01 | 0.65 | 1.06 | 1.89 |
| Heritabilities |  |  |  |  |  |  |
| $h^{2}(T1)$ | 1.01 | 1.01 | 1.60 | 1.57 | 1.49 | 1.92 |
| $h^{2}(T2)$ | 1.00 | 1.00 | 1.00 | 0.68 | 1.15 | 0.84 |
| $h^{2}(T3)$ | 1.00 | 1.01 | 1.01 | 1.28 | 1.29 | 0.55 |
| Additive genetic correlations |  |  |  |  |  |  |
| $r_{a}(T1,T2)$ | 1.01 | 1.01 | 1.71 | 0.29 | 1.35 | 1.65 |
| $r_{a}(T1,T3)$ | 1.01 | 1.03 | 1.14 | 0.96 | 1.02 | 1.31 |
| $r_{a}(T2,T3)$ | 1.00 | 1.01 | 1.02 | 0.55 | 0.70 | 1.19 |
| Residual genetic correlations |  |  |  |  |  |  |
| $r_{e}(T1,T2)$ | 1.00 | 1.00 | 1.01 | 0.92 | 1.55 | 1.67 |
| $r_{e}(T1,T3)$ | 1.00 | 1.01 | 1.01 | 1.03 | 1.15 | 1.51 |
| $r_{e}(T2,T3)$ | 1.00 | 1.00 | 1.01 | 0.70 | 1.05 | 1.87 |
