# Supplementary material for: Performance of the No-U-Turn sampler in multi-trait variance component estimation using genomic data
Source: Genet Sel Evol. 2022 Jul 11;54:51. doi: 10.1186/s12711-022-00743-5 (PMC9275044; doi:10.1186/s12711-022-00743-5)
Supplement: Supplementary file 6 — Additional file 6: Table S4. Gelman and Rubin’s R convergence diagnostic and Geweke’s convergence diagnostic in Scenario 2 for the PIC pig data. [file 12711_2022_743_MOESM6_ESM.docx]

**Table S4** Gelman and Rubin’s R convergence diagnostic and Geweke’s convergence diagnostic in scenario 2 of PIC pig data

| Parameter | Gelman and Rubin’s R convergence diagnostic ($\hat{R}$) | | | Geweke’s convergence diagnostic (z-score) | | |
| --- | --- | --- | --- | --- | --- | --- |
|  | NUTS (LKJ prior) | NUTS (IW prior) | GS | NUTS (LKJ prior) | NUTS (IW prior) | GS |
| Additive (co)variances |  |  |  |  |  |  |
| $\sigma_{a}^{2}(T1)$ | 1.00 | 1.00 | 1.83 | 0.87 | 0.33 | 1.74 |
| $\sigma_{a}^{2}(T2)$ | 1.00 | 1.00 | 1.02 | 0.64 | 1.21 | 1.16 |
| $\sigma_{a}^{2}(T3)$ | 1.00 | 1.02 | 1.02 | 0.78 | 1.02 | 1.34 |
| $\sigma_{a}(T1,T2)$ | 1.02 | 1.02 | 1.37 | 0.62 | 0.94 | 1.12 |
| $\sigma_{a}(T1,T3)$ | 1.01 | 1.00 | 1.25 | 0.47 | 1.26 | 2.18 |
| $\sigma_{a}(T2,T3)$ | 1.00 | 1.00 | 1.04 | 0.75 | 1.06 | 1.10 |
| Residual (co)variances |  |  |  |  |  |  |
| $\sigma_{e}^{2}(T1)$ | 1.00 | 1.00 | 1.05 | 0.97 | 0.10 | 1.48 |
| $\sigma_{e}^{2}(T2)$ | 1.00 | 1.00 | 1.01 | 0.65 | 1.18 | 0.96 |
| $\sigma_{e}^{2}(T3)$ | 1.00 | 1.01 | 1.01 | 0.86 | 0.97 | 1.41 |
| $\sigma_{e}(T1,T2)$ | 1.00 | 1.00 | 1.09 | 0.45 | 1.21 | 1.84 |
| $\sigma_{e}(T1,T3)$ | 1.00 | 1.00 | 1.02 | 0.63 | 1.55 | 2.39 |
| $\sigma_{e}(T2,T3)$ | 1.00 | 1.00 | 1.03 | 0.89 | 0.99 | 0.95 |
| Heritabilities |  |  |  |  |  |  |
| $h^{2}(T1)$ | 1.00 | 1.01 | 1.84 | 0.88 | 0.30 | 1.72 |
| $h^{2}(T2)$ | 1.00 | 1.00 | 1.02 | 0.64 | 1.22 | 1.22 |
| $h^{2}(T3)$ | 1.00 | 1.02 | 1.02 | 0.81 | 1.05 | 1.43 |
| Additive genetic correlations |  |  |  |  |  |  |
| $r_{a}(T1,T2)$ | 1.01 | 1.03 | 2.66 | 0.55 | 0.93 | 2.04 |
| $r_{a}(T1,T3)$ | 1.00 | 1.00 | 2.01 | 0.50 | 1.20 | 2.88 |
| $r_{a}(T2,T3)$ | 1.00 | 1.00 | 1.04 | 0.96 | 0.97 | 1.10 |
| Residual genetic correlations |  |  |  |  |  |  |
| $r_{e}(T1,T2)$ | 1.00 | 1.00 | 1.09 | 0.41 | 1.22 | 1.75 |
| $r_{e}(T1,T3)$ | 1.00 | 1.00 | 1.02 | 0.67 | 1.68 | 2.46 |
| $r_{e}(T2,T3)$ | 1.00 | 1.00 | 1.03 | 0.80 | 0.96 | 0.94 |
