# Supplementary material for: Performance of the No-U-Turn sampler in multi-trait variance component estimation using genomic data
Source: Genet Sel Evol. 2022 Jul 11;54:51. doi: 10.1186/s12711-022-00743-5 (PMC9275044; doi:10.1186/s12711-022-00743-5)
Supplement: Supplementary file 7 — Additional file 7: Table S5. Presence of divergence and parameters that affect divergence for the No-U-Turn Sampler. [file 12711_2022_743_MOESM7_ESM.docx]

**Table S5** Presence of divergence and the parameters affecting divergence for the No-U-Turn Sampler

|  | Simulated data | | | | PIC data | | | |
| --- | --- | --- | --- | --- | --- | --- | --- | --- |
|  | Scenario 1 | | Scenario 2 | | Scenario 1 | | Scenario 2 | |
|  | LKJ prior | IW prior | LKJ prior | IW prior | LKJ prior | IW prior | LKJ prior | IW prior |
| No. divergence | 0 | 0 | 0 | 0 | 0 | 0 | 0 | 0 |
| No. leapfrog step | 60.7 | 63.7 | 42.0 | 36.7 | 51.0 | 52.3 | 42.3 | 38.0 |
| Tree depth | 5.3 | 5.6 | 5.1 | 5.0 | 5.2 | 5.2 | 5.3 | 5.0 |
