# Supplementary material for: Performance of the No-U-Turn sampler in multi-trait variance component estimation using genomic data
Source: Genet Sel Evol. 2022 Jul 11;54:51. doi: 10.1186/s12711-022-00743-5 (PMC9275044; doi:10.1186/s12711-022-00743-5)
Supplement: Supplementary file 8 — Additional file 8: Table S6. Number of iterations and computing time (seconds) for estimating variance components. [file 12711_2022_743_MOESM8_ESM.docx]

**Table S6** Number of iterations and computing time (seconds) for estimating variance components

|  | Simulated data | | | | | | PIC data | | | | | |
| --- | --- | --- | --- | --- | --- | --- | --- | --- | --- | --- | --- | --- |
|  | Scenario 1 | | | Scenario 2 | | | Scenario 1 | | | Scenario 2 | | |
|  | Number of iterations | Computing time | | Number of iterations | Computing time | | Number of iterations | Computing time | | Number of iterations | Computing time | |
|  |  | Per iteration | Total |  | Per iteration | Total |  | Per iteration | Total |  | Per iteration | Total |
| NUTS (LKJ prior) | 2,000 | 7.37 | 14738 | 2,000 | 0.31 | 618 | 2,000 | 7.47 | 14940 | 2,000 | 0.42 | 835 |
| NUTS (IW prior) | 2,000 | 5.96 | 11916 | 2,000 | 0.27 | 536 | 2,000 | 7.16 | 14328 | 2,000 | 0.40 | 791 |
| GS | 10,000 | 0.78 | 7809 | 10,000 | 0.07 | 679 | 10,000 | 1.02 | 10188 | 10,000 | 0.08 | 771 |
| REML | 16 | 85.43 | 1367 | 17 | 1.52 | 26 | 11 | 337.63 | 3714 | 75 | 5.53 | 415 |
